# Prediction of Clinical Outcome in Locally Advanced Non-Small Cell Lung Cancer Patients Treated With Chemoradiotherapy by Plasma Markers

**DOI:** 10.3389/fonc.2020.625911

**Published:** 2021-02-17

**Authors:** Xin Sui, Leilei Jiang, Huajing Teng, Lan Mi, Bo Li, Anhui Shi, Rong Yu, Dongming Li, Xin Dong, Dan Yang, Huiming Yu, Weihu Wang

**Affiliations:** ^1^ Key Laboratory of Carcinogenesis and Translational Research (Ministry of Education/Beijing), Department of Radiation Oncology, Peking University Cancer Hospital and Institute, Beijing, China; ^2^ Key Laboratory of Carcinogenesis and Translational Research (Ministry of Education/Beijing), Department of Lymphoma, Peking University Cancer Hospital and Institute, Beijing, China

**Keywords:** non-small cell lung cancer, chemoradiotherapy, cytokines, IL-8, intercellular adhesion molecule 1 (ICAM-1), biomarkers

## Abstract

**Purpose:**

To identify cytokines in plasma that may predict objective response and progression-free survival (PFS) in patients with locally advanced non-small cell lung cancer (NSCLC) treated with chemoradiotherapy.

**Materials and Methods:**

From April 2016 to May 2017, thirty-one patients with locally advanced inoperable/unresectable NSCLC were included, and treated with concurrent chemoradiotherapy (CCRT). No immune checkpoint inhibitors were administered after CCRT. Plasma from each patient was collected before radiotherapy, and 25 cytokines in the plasma were measured by Luminex or U-PLEX assays. Logistic regression and COX regression were performed to identify the predictive factors for objective response and PFS, respectively. Kaplan-Meier survival analysis was used to compare the PFS between the groups.

**Results:**

High levels of IL-13 and TNF-α, and low levels of ICAM-1, IFN-γ, and soluble PD-L1 (sPD-L1) were significantly associated with objective response (*P <*0.05). High levels of IL-8, CCL5, and CXCL3 also showed a trend toward association with objective response (*P <*0.1). The combination of cytokines (IL-8 and ICAM-1, or TNF-α and sPD-L1) improved predictive accuracy. Univariate analysis identified IL-8 and ICAM-1 as potential markers to predict PFS. Multivariate analysis suggested that high level of IL-8 (*P* =0.010) and low level of ICAM-1 (*P* =0.011) correlated significantly with a longer PFS.

**Conclusion:**

IL-8 and ICAM-1 in plasma have the potential to predict objective response and PFS in patients with locally advanced NSCLC underwent chemoradiotherapy.

## Introduction

Locally advanced non-small cell lung cancer (NSCLC) accounts for approximately 30% of NSCLC cases. Definitive concurrent chemoradiotherapy (CCRT) is the standard treatment for patients with unresectable or inoperable locally advanced NSCLC (1, 2). Concurrent regimen with once-daily thoracic radiation improves the 5-year survival compared with sequential chemoradiotherapy or concurrent regimen with twice-daily hyperfractionated radiotherapy (2). However, the outcome of radiotherapy varies among patients. The response rate varies between different chemotherapy regimens, from approximately 38% to 80% (3–5). The median progression-free survival (PFS) of concurrent chemoradiotherapy in locally advanced NSCLC is about 8–15 months (3–5). For patients whose tumor relapse or metastasis are likely to occur, more aggressive treatment or more frequent follow-up may be needed. Therefore, prediction of the outcome of radiotherapy is urgently needed for the selection of an optimal treatment strategy.

Cytokines and other soluble factors participate in carcinogenesis and treatment response. Factors that are involved in inflammatory and immune responses may contribute to tumor control and survival (6). Suwinski et al. measured six serum proteins in NSCLC patients treated with curative or palliative thoracic radiotherapy, and demonstrated potential utilities of osteopontin (OPN), vascular endothelial growth factor (VEGF), and erythropoietin (EPO) as prognostic factors (7). Using blood-biomarkers related to hypoxia, inflammation and tumor load, Carvalho et al. developed a model to predict survival of NSCLC patients receiving radiotherapy (8). These studies provided valuable information for noninvasively predicting clinical outcome for NSCLC patient treated with radiotherapy. However, the populations were relatively heterogeneous, including patients with stage I inoperable tumor, or patients with metastatic receiving palliative radiotherapy. Therefore the results may not be directly translated into the prediction of response to CCRT for patients with locally advanced NSCLC.

To identify peripheral blood-based biomarkers that may predict the objective response and PFS in patients with locally advanced NSCLC treated with chemoradiotherapy, we collected plasma from these patients prior to radiotherapy, and detected a panel of cytokines or soluble factors, which involve in inflammation, immune status, angiogenesis and other biological processes. We identified candidate biomarkers which have the potential to predict the objective response and PFS in patients with locally advanced NSCLC underwent chemoradiotherapy.

## Materials and Methods

### Patients and Treatment

From April 2016 to May 2017, 31 patients with locally advanced inoperable/unresectable NSCLC were continuously included in the study, and treated with CCRT in the Department of Radiation Oncology, Peking University Cancer Hospital and Institute, China. Radiation was delivered using the intensity-modulated radiotherapy (IMRT) technique, and a total dose of 60–66 Gy was administered in 2.0–2.2 Gy daily fractions over 6 weeks using 6 MV photons. Concurrent chemotherapy included pemetrexed/carboplatin (for adenocarcinoma), docetaxel/carboplatin, paclitaxel/carboplatin, etoposide/carboplatin, or paclitaxel. The majority of patients received platinum-based induction chemotherapy before CCRT. No immune checkpoint inhibitors were administered after CCRT. Patients were followed up approximately every 3 months during the first year after radiotherapy, every 6 months during the second year, and annually thereafter. The study protocol was approved by the Ethics Committee of Beijing Cancer Hospital (Peking University Cancer Hospital and Institute). Each patient provided written informed consent.

### Sample Collection and Cytokine Measurement

Blood samples were collected into EDTA-containing collection tubes from each patient within 3 days before the beginning of radiotherapy, and from 17 patients in this cohort at the end of radiotherapy. Blood samples were centrifuged within 1 h of collection at 1000 × g for 20 min at 4°C. The supernatant was collected as plasma and was stored at -80°C until analysis.

Interleukin-2 (IL-2), IL-10, IL-12p70, vascular endothelial growth factor (VEGF), tumor necrosis factor-related apoptosis-inducing ligand (TRAIL), chemokine (C-X-C motif) ligand 3 (CXCL3), chemokine (C-C motif) ligand 5 (CCL5), colony-stimulating factor (M-CSF), granulocyte colony-stimulating factor (G-CSF), thrombopoietin (TPO), erythropoietin (EPO), WNT3A, soluble programmed death-ligand 1 (sPD-L1), interferon gamma (IFN-γ), and intercellular adhesion molecule 1 (ICAM-1) were measured by Luminex assay (R&D Systems, Minneapolis, MN). Eotaxin, FMS-related tyrosine kinase 3 ligand (FLT3L), granulocyte-macrophage colony stimulating factor (GM-CSF), IL-1α, IL-1β, IL-6, IL-8, IL-13, IP-10, and tumor necrosis factor-alpha (TNF-α) were measured by U-PLEX assay (Meso Scale Diagnostics, Rockville, MD, USA).

### Statistics Analysis

Objective response rate (ORR) was defined as the percentage of patients with the best overall response of complete remission (CR) and partial remission (PR) according to Response Evaluation Criteria In Solid Tumors version 1.1 (RECIST1.1). PFS was defined as the time from the first day of radiotherapy to the date of any progression, death, and loss to follow-up. Patients were censored at the last follow-up if no progression or death occurred. Logistic regression was performed to identify the predictive factors for objective response. The area under the curve (AUC) of the receiver operating characteristic (ROC) curves was calculated for the prediction of objective response. COX regression was performed to identify the predictive factors for PFS. ROC analysis was applied to optimize the cut-off. Kaplan-Meier survival curves were applied to compare the PFS between the groups. Variables with *P*<0.1 under univariate analysis were selected for multivariate analysis. *P* values <0.05 (two-sided) were considered statistically significant. All statistical analyses were performed using SPSS Statistics 21.0 (IBM SPSS Statistics, Chicago, IL).

## Results

### Patient Characteristics

From April 2016 to May 2017, there were 31 patients included in the analysis. Patient characteristics are shown in **Table 1**. The median age was 58.4 years (range: 43.3–69.5). The median follow-up time was 33.9 months (95% CI: 28.6–39.2). The percentage of patients with partial remission (PR), stable disease (SD), and progression disease (PD) was 58.1%, 32.3%, and 9.7%, respectively. The ORR was 58.1%, and 23 patients (74.2%) experienced disease progression at the last follow-up. Median PFS was 10.5 months (95% CI: 4.4–16.7), and the 1-year and 2-year PFS were 48.4% and 25.8% respectively. Median overall survival (OS) was not reached, and the 1-year and 2-year OS were 87.1% and 70.1% respectively.

**Table 1:** Patient characteristics (N=31).

Characteristic	N	%
Age, median years (range)	58.4 (43.3–69.5)	
Gender		
Male	26	83.9
Female	5	16.1
Smoking		
Never	6	19.4
Ever	25	80.6
Histology		
Adenocarcinoma (ADC)	7	22.6
Squamous cell carcinoma (SCC)	24	77.4
Stage		
IIB	1	3.2
IIIA	10	32.3
IIIB	20	64.5
EGFR mutation		
Wildtype	28	90.3
Mutant (Del19/L858R/T2573G)	3 (1/1/1)	9.7 (3.2/3.2/3.2)
Induction chemotherapy		
No	5	16.1
Yes	26	83.9

### Correlation Between Objective Response and Cytokines in Plasma

We analyzed the association between objective response and clinical factors or cytokines in plasma collected prior to CCRT by logistic regression (**Table 2**). Pathology, EGFR mutation status and induction chemotherapy were not associated with objective response. Patients with objective response to CCRT had high levels of IL-13 and TNF-α, and low levels of ICAM-1, IFN-γ, and sPD-L1. High levels of IL-8, CCL5, and CXCL3 also showed a trend toward association with objective response.

**Table 2:** Association between objective response and clinical factors or cytokines in plasma by univariate analysis.

Factor	Median	Odds Ratio (95% CI)	*P*
Age (years)	58.3	1.129 (0.987–1.291)	0.076
Gender (Male vs. Female)	NA	0.417 (0.059–2.946)	0.38
Smoking (Never vs. Ever)	NA	1.5 (0.251–8.977)	0.657
Pathology (ADC vs. SCC)	NA	2.222 (0.402–12.285)	0.36
Stage* (IIB vs. IIIA vs. IIIB)	NA	0.371 (0.084–1.638)	0.191
EGFR mutation (wildtype vs. mutant)	NA	0.000 (NA)	0.999
Induction chemotherapy (No vs. Yes)	NA	0.909 (0.129–6.396)	0.924
**CCL5 (ng/ml)**	**14.863**	**1.051 (0.998–1.107)**	**0.062**
**CXCL3**	**314.12**	**1.004 (0.999–1.008)**	**0.096**
Eotaxin	139.09	1.005 (0.992–019)	0.445
EPO	15.455	1.042 (0.956–1.136)	0.345
FLT3L	175.11	1 (0.994–1.005)	0.942
G-CSF	23.59	0.984 (0.954–1.015)	0.321
GM-CSF	0.18	0.307 (0.000–3465.908)	0.804
**ICAM-1 (ng/ml)**	**553.397**	**0.997 (0.995–1.000)**	**0.03**
**IFN-γ**	**25.36**	**0.963 (0.928–0.998)**	**0.038**
IL-1α	0.51	1.084 (0.677–1.736)	0.737
IL-1β	0.2	1.533 (0.007–350.442)	0.878
IL-10	1.3	1.378 (0.056–33.828)	0.844
IL-12p70	40.9	0.933 (0.804–1.083)	0.362
**IL-13**	**3.2**	**1.421 (1.039–1.941)**	**0.028**
IL-2	5.7	0.496 (0.155–1.593)	0.239
IL-6	1.73	1.114 (0.832–1.491)	0.469
**IL-8**	**4.12**	**1.507 (0.964–2.355)**	**0.072**
IP-10	414.65	1.002 (0.997–1.006)	0.474
M-CSF	50.11	0.993 (0.971–1.016)	0.57
**sPD-L1**	**68.27**	**0.997 (0.995–1.000)**	**0.042**
**TNF-α**	**4.59**	**2.985 (1.230–7.242)**	**0.016**
TPO	28.54	0.999 (0.994–1.004)	0.661
TRAIL	31.57	1.007 (0.971–1.044)	0.702
VEGF	16.1	1.061 (0.987–1.141)	0.108
WNT3A	0.751	0.641 (0.172–2.396)	0.509

The unit of the cytokines is pg/ml if not otherwise specified. Cytokines with P<0.1 are shown in bold.

*According to American Joint Commission on Cancer (AJCC) staging system 7^th^ edition.

**Figure 1:**
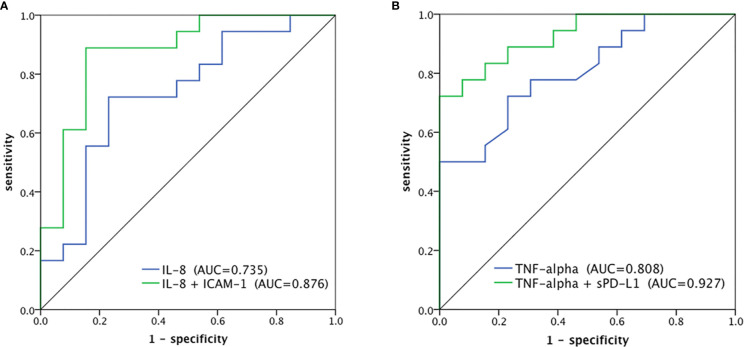
ROC curves to predict objective response of CCRT. **(A)** IL8 alone and IL-8 in combination with ICAM-1, **(B)** TNF-α alone and TNF-α in combination with sPD-L1.

Multivariate logistic analysis showed that the combination of IL-8 and ICAM-1, or sPD-L1 and TNF-α, might predict the objective response to CCRT (**Table 3**). ROC curve analysis was performed to assess whether the levels of cytokines would predict objective response to chemoradiotherapy. The AUC was 0.744 (95% CI: 0.567–0.920; *P*=0.022) with ICAM-1, 0.735 (95% CI: 0.551–0.919; *P*=0.028) with IL-8, 0.808 (95% CI: 0.658–0.958; *P*=0.004) with TNF-α, and 0.774 (95% CI: 0.598–0.949; *P*=0.010) with sPD-L1. The combination of cytokines improved the prediction accuracy. The combination of IL-8 and ICAM-1 enhanced the AUC to 0.876 (95% CI: 0.745–1.000; *P*=0.000) (**Figure 1A**). The combination of sPD-L1 and TNF-α provided a higher AUC of 0.927 (95% CI: 0.842–1.000; *P*=0.000) (**Figure 1B**). The AUC was not improved by the other combinations of cytokines.

**Table 3:** Association between objective response and cytokines in plasma by multivariate analysis.

	Cytokine	Odds Ratio (95% CI)	*P*
Combination 1	IL-8	1.975 (1.004–885)	0.049
	ICAM-1	0.996 (0.994–0.999)	0.017
			
Combination 2	sPD-L1	0.996 (0.992–0.999)	0.014
	TNF-α	5.060 (1.380–18.553)	0.014

Cytokines in plasma collected from 17 patients at the end of radiotherapy were also measured and compared with pre-CCRT levels with paired t-test. Levels of CXCL3, EPO, FLT3L, GM-CSF, IL-1α, IL-1β, IL-6, IP-10, TNF-α, TRAIL, and TPO significantly increased after CCRT. Other cytokines showed no significant difference between pre- and post-CCRT. Neither cytokine levels post CCRT nor the post-/pre-CCRT ratios of cytokines were associated with objective response. However, the sample size was too small to reach a conclusion.

### Correlation Between PFS and Cytokines in Plasma

**Figure 2:**
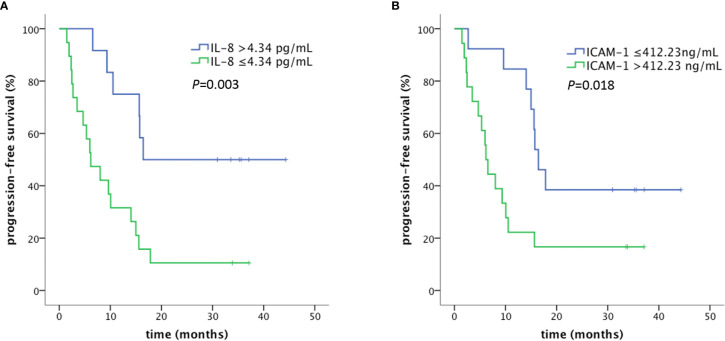
PFS in patients with high and low levels of plasma IL-8 **(A)** and ICAM-1 **(B)**. PFS was defined as the time from the first day of radiotherapy to the date of any progression, death, and loss to follow-up. Patients were censored at the last follow-up if no progression or death occurred.

The association between PFS and clinical factors or cytokines in plasma in prior to CCRT was analyzed by univariate Cox regression (**Table 4**). Patients who achieved objective response after chemoradiotherapy had longer PFS (*P*=0.006). The median PFS was 15.6 months (95% CI: 14.0–17.1) and 5.3 months (95% CI: 1.5–9.2) in patients with and without objective response, respectively. Pathology, EGFR mutation status and induction chemotherapy were not correlated with PFS. The level of IL-8 was correlated with PFS (HR=0.788, *P*=0.036). Using the cut-off optimized by ROC analysis, patients with high levels of plasma IL-8 (>4.34 pg/ml) had a longer median PFS (16.4 months, 95% CI: NA) compared with 6.2 months (95% CI: 2.4–10.0) in the low IL-8 (≤4.34 pg/ml) group (**Figure 2A**). Although not significant, the level of ICAM-1 also showed a trend toward association between lower concentration and longer PFS (HR=1.001, *P*=0.07). The median PFS of patients with high level of ICAM-1 (>412.23 ng/ml) and low level of ICAM-1 (≤412.23 ng/ml) was 6.2 months (95% CI: 4.9–7.3) and 16.4 months (95% CI: 13.8–19.1), respectively (**Figure 2B**).

**Table 4:** Association between PFS and clinical factors or cytokines in plasma by univariate analysis.

Factor	Hazard Ratio (95% CI)	*P*
Age (years)	1.021 (0.960–1.085)	0.513
Gender (Male vs. Female)	2.511 (0.908–6.948)	0.076
Smoking (Never vs. Ever)	1.138 (0.428–3.022)	0.796
Pathology (ADC vs. SCC)	0.596 (0.245–1.453)	0.255
Stage* (IIB vs. IIIA vs. IIIB)	1.679 (0.750–3.755)	0.207
EGFR mutation (wildtype vs. mutant)	2.212 (0.622–7.861)	0.220
Induction chemotherapy (No vs. Yes)	0.606 (0.238–1.542)	0.293
CCL5	0.987 (0.961–1.015)	0.357
CXCL3	0.999 (0.997–1.000)	0.121
Eotaxin	0.999 (0.991–1.007)	0.812
EPO	1.005 (0.984–1.027)	0.651
FLT3L	0.997 (0.993–1.002)	0.218
G-CSF	1.009 (0.993–1.026)	0.284
GM-CSF	0.465 (0.002–109.226)	0.783
**ICAM-1**	**1.001 (1.000–1.001)**	**0.070**
IFN-γ	1.011 (0.996–1.027)	0.143
IL-1α	1.001 (0.766–1.310)	0.992
IL-1β	4.388 (0.165–117.024)	0.377
IL-10	0.225 (0.036–1.395)	0.109
IL-12p70	0.952 (0.879–1.032)	0.235
IL-13	0.979 (0.855–1.121)	0.759
IL-2	1.149 (0.680–1.940)	0.605
IL-6	0.985 (0.845–1.147)	0.845
**IL-8**	**0.788 (0.630–0.985)**	**0.036**
IP-10	0.998 (0.995–1.001)	0.152
M-CSF	1 (0.989–1.010)	0.953
sPD-L1	1.001 (1.000–1.002)	0.117
TNF-α	0.974 (0.710–1.336)	0.869
TPO	0.999 (0.996–1.002)	0.550
TRAIL	0.984 (0.963–1.006)	0.155
VEGF	0.978 (0.947–1.009)	0.155
WNT3A	1.333 (0.589–3.020)	0.491

Cytokines with P<0.1 are shown in bold. *According to American Joint Commission on Cancer (AJCC) staging system 7^th^ edition.

**Figure 3:**
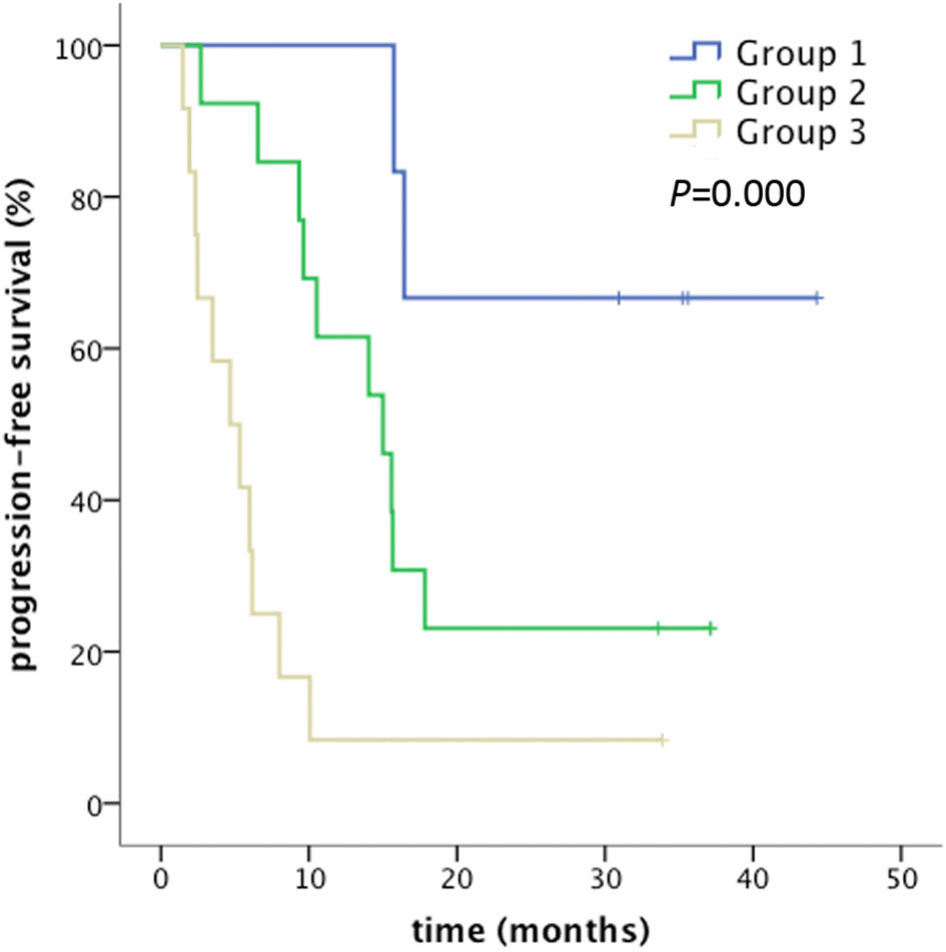
Combination effect of IL-8 and ICAM-1 for PFS prediction. Group 1: IL-8 >4.34 pg/ml, ICAM-1 ≤412.23 ng/ml; Group 2: IL-8 >4.34 pg/ml, ICAM-1 >412.23 ng/ml or IL-8 ≤4.34 pg/ml, ICAM-1 ≤412.23 ng/ml; Group 3: IL-8 ≤4.34 pg/ml, ICAM-1 >412.23 ng/ml. PFS was defined as the time from the first day of radiotherapy to the date of any progression, death, and loss to follow-up. Patients were censored at the last follow-up if no progression or death occurred.

IL-8 and ICAM-1 were selected for multivariate analysis (**Table 5**). High level of IL-8 and low level of ICAM-1 correlated significantly with longer PFS. Patients were stratified into three groups according to the levels of IL-8 and ICAM-1 concentration in plasma: Group 1 with high IL-8 and low ICAM-1; Group 2 with high IL-8 and high ICAM-1, or low IL-8 and low ICAM-1; and Group 3 with low IL-8 and high ICAM-1 (**Figure 3**). The median PFS in Group 1 was not reached, and all six patients in this group were alive at the last follow-up. The median PFS was 15.0 months (95% CI: 9.1-21.0) in Group 2, and 4.7 months (95% CI: 1.6-7.8) in Group 3 (*P*=0.000).

**Table 5:** Association between PFS and cytokines in plasma by multivariate analysis.

Cytokine	Hazard Ratio (95% CI)	*P*
IL-8	0.716 (0.555–0.925)	0.010
ICAM-1	1.001 (1.000–1.002)	0.011

## Discussion

This study systemically investigated the value of cytokines in plasma as a predictive marker for clinical outcome in patients with locally advanced NSCLC treated with chemoradiotherapy. High levels of IL-13, TNF-α, IL-8, CCL5, and CXCL3, and low levels of ICAM-1, IFN-γ, and sPD-L1 before radiotherapy were associated (or showed a trend toward association) with objective response after chemoradiotherapy. The combination of IL-8 and ICAM-1, or sPD-L1 and TNF-α, improved the prediction accuracy of objective response with a higher AUC. Only the levels of IL-8 and ICAM-1 translated into the prediction of PFS. Patients with high IL-8 and low ICAM-1 had longer PFS. This study provided candidate markers for the prediction of response and PFS of chemoradiotherapy in patients with locally advanced NSCLC.

IL-8, also known as CXCL8, has been reported as a marker for poor outcomes in hormone-dependent breast cancer (9), colorectal cancer (10, 11), and lung cancer (12, 13). Xiao et al. reported that high CXCL8 immunohistochemical staining score was associated with shorter OS and disease-free survival (DFS) in patients with colorectal cancer (8). Liu et al. summarized the expression of CXCL8 in different stages of NSCLC. High CXCL8 mRNA levels were associated with advanced stage and poor survival in NSCLC (12). Ryan et al. reported that high levels of serum IL-8 were associated with an increased risk of lung cancer mortality in patients with Stage I lung cancer (13). In our dataset, plasma IL-8 prior to radiotherapy was not correlated with OS (*P*=0.832, data not shown), but correlated with objective response and PFS of chemoradiotherapy. Sanmamed et al. reported that the dynamics of serum IL-8 levels predict the response to anti-programmed cell death protein 1 (PD-1) treatment. In responders, IL-8 levels decreased between baseline and best response, and increased upon progression (14). In our cohort, the change in IL-8 level was not associated with objective response in 17 patients whose blood samples were collected before and after CCRT. However, the population was too limited to draw conclusions. IL-8 binds to C-X-C motif chemokine receptor 1 and 2 (CXCR1 and CXCR2), and activates the phosphatidyl-inositol-3-kinase (PI3K) signaling pathway, leading to the activation of downstream signals including Akt, protein kinase C (PKC), mitogen-activated protein kinase (MAPK), etc. (15). It has been reported that IL-8 could enhance the production of matrix metalloproteinases in CXCR1- and CXCR2-expressing endothelial cells and regulate angiogenesis (16). It is possible that IL-8 promoted angiogenesis and reduced hypoxia in tumors, thereby increasing the radiosensitivity of tumor cells.

ICAM-1 belongs to the immunoglobulin gene superfamily. It is involved in the inflammatory process and tumor metastasis. High levels of ICAM-1 were correlated with advanced stage and poor prognosis in multiple types of cancer (17–21). Qian et al. reported that advanced NSCLC patients with lower levels of soluble ICAM-1 had longer survival and a higher objective response rate of chemotherapy (17). Zhou et al. collected exhaled breath condensate (EBC) and peripheral blood samples from patients with NSCLC, patients with chronic obstructive pulmonary disease (COPD) and healthy controls, and detected the level of soluble ICAM-1. sICAM-1 was significantly elevated in both EBC and serum in patients with NSCLC compared to that in patients with COPD and healthy controls, and high levels of both exhaled and serum sICAM-1 were associated with distant metastasis (18). This suggested the role of ICAM-1 in diagnosis and prognosis was both local and systemic. In our study, lower plasma ICAM-1 before chemoradiotherapy was correlated with objective response and longer PFS, which was in accordance with the prognostic value of ICAM-1 in previous studies.

We also identified high level of TNF-α and low level of sPD-L1 as plasma markers for predicting objective response to chemoradiotherapy in locally advanced NSCLC. TNF-α is an inflammatory cytokine, and its role in cancer is complicated. Endogenous TNF-α has tumor-promoting activity. The level of TNF-α was elevated in cancers compared with normal tissues or healthy controls (22). Meanwhile, other studies have reported that TNF-α also has anti-tumor activity by destroying tumor vasculature and facilitating antitumor immune response (23). Boldrini et al. reported that TNF-α mRNA levels in surgically resected cancer tissues was associated with better prognosis in NSCLC (24). PD-L1 binds to PD-1, resulting in T cell dysfunction. Blockade of PD-1/PD-L1 is applied to many types of malignancies (25). Meta-analysis showed that NSCLC patients with positive immunohistochemical PD-L1 staining had poor OS (26). Soluble PD-L1 has been reported to be associated with poor prognosis in lung cancer and hepatocellular carcinoma (27–29). Our results suggested that plasma TNF-α was positively correlated with objective response, while sPD-L1 was negatively correlated with objective response to chemoradiotherapy. The combination of these two factors enhanced the predictive accuracy (AUC=0.927). However, the predictive value of the objective response did not translate into the prediction of PFS.

Our study had several limitations. First, the sample size was limited. The identified potential markers need to be validated in a larger population. Despite the limited population, patients in our cohort received concurrent definitive CCRT, and the radiotherapy is delivered using IMRT technique. The modality of treatment was relatively homogenous, which enhanced the stringency of this study. Second, the rationale for prediction of objective response and PFS needs to be explored. The dynamics of cytokines during treatment may provide clues to understand the relationship between cytokines and clinical outcomes. However, some post-treatment blood samples were not available in this study. We will compare the levels of cytokines before and after radiotherapy in further studies. Third, although adding induction chemotherapy to CCRT could not bring benefits according to a randomized phase 3 trial (30), most of the patients in our study received induction chemotherapy prior to CCRT due to the pattern of referrals in our region. Blood samples were collected before CCRT, and PFS was calculated from the first day of radiotherapy. There was no significant difference in objective response or PFS between patients with or without induction chemotherapy. Fourth, patients were enrolled from April 2016 to May 2017, prior to the publication of PACIFIC results (31), which established the consolidation therapy with durvalumab after CCRT as standard treatment in NCCN guideline. Therefore in this study, no immune checkpoint inhibitors were used after CCRT. Considering durvalumab is used as consolidative rather than concurrent regimen, we speculated the results obtained in this study might provide clues to predict the outcome of CCRT in combination with consolidative PD-L1 blockade. Moreover, due to the medical insurance reasons, durvalumab is not broadly used after CCRT for locally advanced NSCLC currently in China. Therefore, the results still showed some significances under current clinical practices.

In conclusion, cytokines in plasma may predict the response and PFS in patients with locally advanced NSCLC underwent chemoradiotherapy. High levels of TNF-α and IL-8, and low levels of ICAM-1 and sPD-L1 before radiotherapy were associated (or showed a trend toward association) with objective response after chemoradiotherapy. Patients with high IL-8 and low ICAM-1 had longer PFS. Our results provide a potential model to predict the response and PFS of CCRT for patients with NSCLC.

## Data Availability Statement

The original contributions presented in the study are included in the article/supplementary material. Further inquiries can be directed to the corresponding authors.

## Ethics Statement

The studies involving human participants were reviewed and approved by Ethics Committee of Peking University Cancer Hospital and Institute. The patients/participants provided their written informed consent to participate in this study.

## Author Contributions

XS, LJ, HY, and WW: conceptualization. XS, LJ, HT, and LM: methodology. XS, LJ, BL, XD, and DY: investigation. AS, RY, DL, and HY: resources. XS and LJ: writing-original draft preparation. HT, LM, HY, and WW: writing-review and editing. All authors contributed to the article and approved the submitted version.

## Funding

This study was supported by funding from National Natural Science Foundation of China (81672969, 81472814, 82073333, 32071156), Beijing Municipal Science & Technology Commission (Z181100001718192), and Science Foundation of Peking University Cancer Hospital (18-03).

## Conflict of Interest

The authors declare that the research was conducted in the absence of any commercial or financial relationships that could be construed as a potential conflict of interest.
